# Selective and Recyclable Congo Red Dye Adsorption by Spherical Fe_3_O_4_ Nanoparticles Functionalized with 1,2,4,5-Benzenetetracarboxylic Acid

**DOI:** 10.1038/s41598-019-57017-2

**Published:** 2020-01-10

**Authors:** Sobhan Chatterjee, Nikita Guha, Sarathkumar Krishnan, Amrendra K. Singh, Pradeep Mathur, Dhirendra K. Rai

**Affiliations:** 1 Discipline of Chemistry, Indian Institute of Technology Indore, Simrol, Indore, 453552 India; 2 Discipline of Metallurgy Engineering and Materials Science, Indian Institute of Technology Indore, Simrol, Indore, 453552 India

**Keywords:** Pollution remediation, Pollution remediation

## Abstract

In this study, the new material Fe_3_O_4_@BTCA has been synthesized by immobilization of 1,2,4,5-Benzenetetracarboxylic acid (BTCA) on the surface of Fe_3_O_4_ NPs, obtained by co-precipitation of FeCl_3_.6H_2_O and FeCl_2_.4H_2_O in the basic conditions. Characterization by P-XRD, FE-SEM, and TEM confirm Fe_3_O_4_ has a spherical crystalline structure with an average diameter of 15 nm, which after functionalization with BTCA, increases to 20 nm. Functionalization also enhances the surface area and surface charge of the material, confirmed by BET and zeta potential analyses, respectively. The dye adsorption capacity of Fe_3_O_4_@BTCA has been investigated for three common dyes; Congo red (C.R), Methylene blue (M.B), and Crystal violet (C.V). The adsorption studies show that the material rapidly and selectively adsorbs C.R dye with very high adsorption capacity (630 mg/g), which is attributed to strong H-bonding ability of BTCA with C.R dye as indicated by adsorption mechanism study. The material also shows excellent recyclability without any considerable loss of adsorption capacity. Adsorption isotherm and kinetic studies suggest that the adsorption occurs by the Langmuir adsorption model following pseudo-second-order adsorption kinetics.

## Introduction

Organic dyes are one of the significant contributors to water pollution caused by the discharge of effluent from various industries such as textile, plastic, printing, photographic, paper-pulp, paint, and leather^1–9^. There are many techniques for wastewater treatment, for instance, coagulation, electrocoagulation, chlorination, ozonation, flotation, chemical oxidation, filtration, membrane separation, adsorption, and ultrafiltration^10–17^. Among various reported techniques, the adsorption is the most convenient and well-established technique because of high efficiency, simplicity, and minimum energy requirement^18–24^.

In recent times, porous metal oxides have been recognized as excellent solid adsorbents for wastewater treatment owing to their high surface area and the presence of sufficient active surface sites^25–28^. Among various metal oxides, Fe_3_O_4_ nanoparticles (NPs) are drawing considerable attention due to their unique properties such as excellent biocompatibility, low synthetic cost, ease of functionalization, and high magnetic susceptibility leading to easy magnetic recovery^29–34^. Owing to the negatively charged surface, bare Fe_3_O_4_ NPs have been shown as dye absorbents in many reports^35–37^; however, its applicability is limited due to low adsorption capacity and poor selectivity and recyclability. Chaudhary *et al*. have reported the use of Fe_3_O_4_ NPs for adsorption of acridine orange dye, Coomassie brilliant blue R-200 dye, and Congo red dye^37^. Jia *et al*. developed 3D hierarchical porous Fe_3_O_4_ NPs, which have been used for C.R dye adsorption, however, with low adsorption capacity of 39.10 mg/g^25^. Similarly, hydrothermal preparation of Fe_3_O_4_ NPs and its application for C.R dye removal from aqueous solution, with adsorption capacity of 28.46 mg/g, has been reported by Wang *et al*.^35^.

Besides electrostatic interactions as applicable to the cases mentioned above, the adsorption of dye is also actively facilitated by hydrogen bonding, which depends on functional groups and active coordination sites present on the solid surface. Thus, to achieve higher adsorption capacity, many researchers have focused their efforts on the functionalization of the Fe_3_O_4_ surface by introducing organic chelate groups^38–42^ and metal oxide^43–47^. For example, polyaniline (PANI) and carbon nanotube (CNT) functionalized Fe_3_O_4_ NPs were reported by Zhao *et al*., which showed a moderate value of adsorption capacity (417.38 mg/g)^40^. Wang *et al*. reported polyethyleneimine/graphene oxide/Fe_3_O_4_ based nanocomposite material with 574.7 mg/g adsorption capacity^38^.

**Figure 1:**
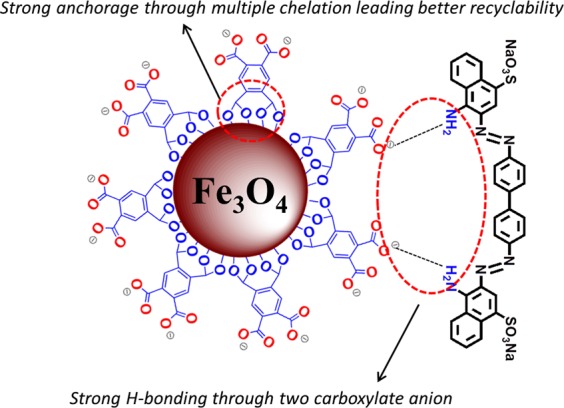
Schematic illustration of Fe_3_O_4_@BTCA material adsorption towards Congo red dye through H-bonding carboxylate anions.

In the present work, we have explored a multi carboxylate organic ligand 1,2,4,5-Benzentetracarboxylic acid (BTCA) for surface functionalization of Fe_3_O_4_ nanoparticles for rapid and selective adsorption of a ubiquitous industrial pollutant Congo red dye. Though there are many reports on surface functionalization with carboxylate groups^48–50^, BTCA group, containing four carboxylate groups at 1, 2, 4 and 5- positions of the benzene ring as functionalization agent, is expected to significantly improve the adsorption capacity for selective dyes with better recyclability as illustrated in Fig. 1. Formation of two covalent bonds, in chelate fashion, by condensation of two –COOH groups from BTCA and two –OH groups from Fe_3_O_4_ surface will cause a firm anchorage of BTCA on Fe_3_O_4_ surface. The remaining two –COO^−^ groups, formed under synthetic conditions, can form a strong H-bonding with a primary amine-containing dye, like Congo red, which will lead to high adsorption capacity. To best of our knowledge, it is the first report where BTCA has been employed as a surface functionalization ligand for any material.

The present report discusses the synthesis and characterization of BTCA functionalized Fe_3_O_4_ NPs (Fe_3_O_4_@BTCA), which rapidly and selectively adsorbs a primary amine-containing Congo red dye from its aqueous solution. The adsorption studies show that Fe_3_O_4_@BTCA material exhibits significantly higher adsorption capacity and can be reused in multiple cycles without undergoing any considerable loss in adsorption performance. Mechanisms for selective adsorption and adsorption kinetics have also been described.

## Results and Discussion

**Figure 2:**
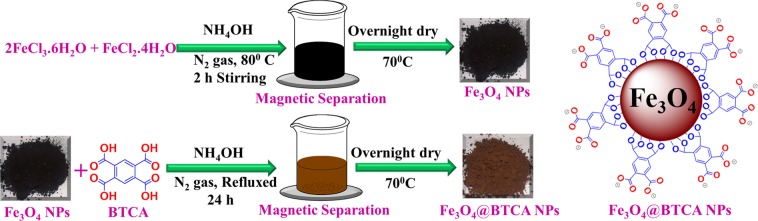
Schematic illustration of the preparation of surface-modified Fe_3_O_4_@BTCA.

**Figure 3:**
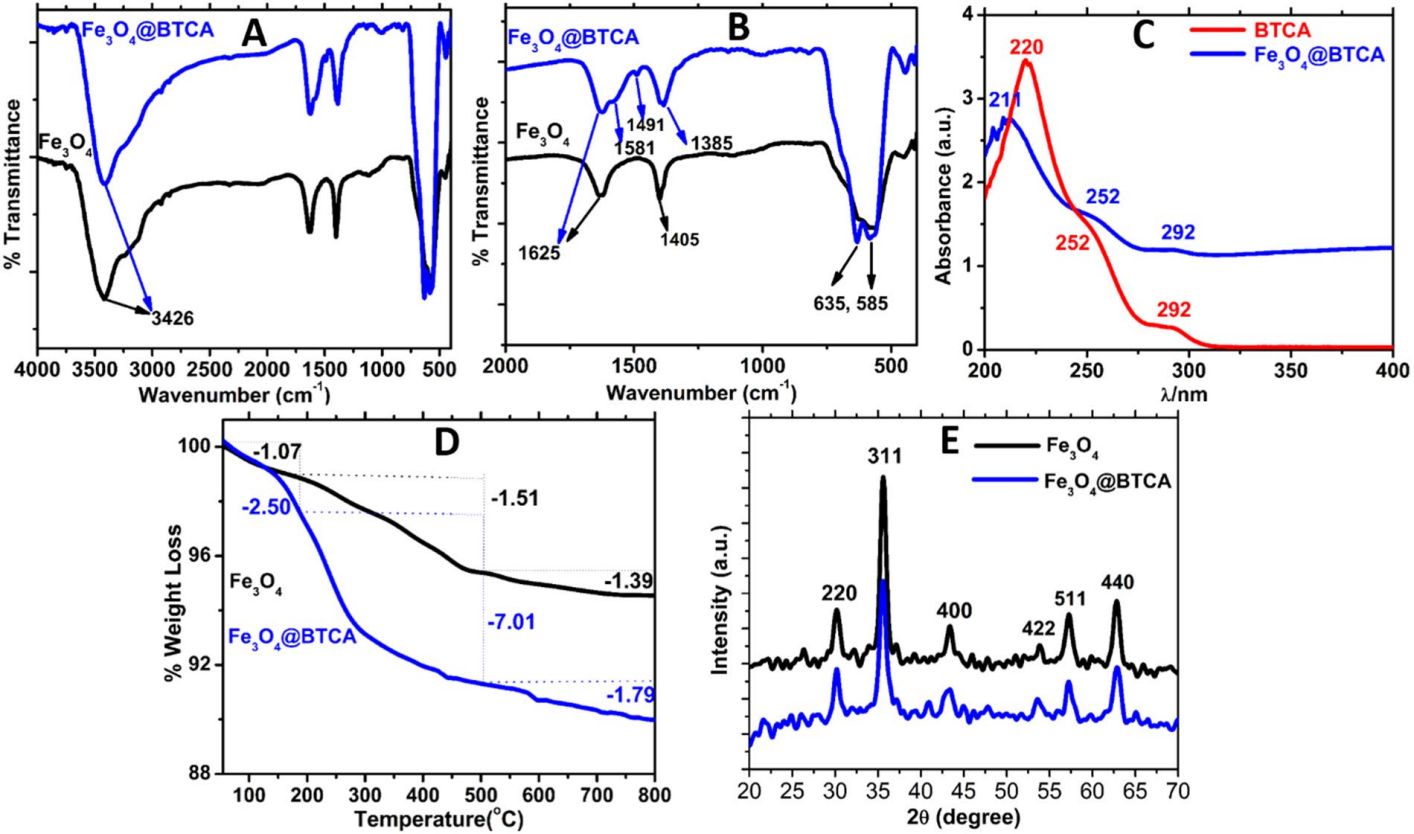
(**A**) FT-IR spectra of Fe_3_O_4_ and Fe_3_O_4_@BTCA, (**B**) FT-IR for shorter range, (**C**) UV-Vis spectra of BTCA and Fe_3_O_4_@BTCA, (**D**) TGA and (**E**) PXRD pattern of Fe_3_O_4_ and Fe_3_O_4_@BTCA.

The synthetic steps involved in the preparation of Fe_3_O_4_ and subsequent immobilization of BTCA groups to obtain Fe_3_O_4_@BTCA is depicted in Fig. 2. To confirm the formation of Fe_3_O_4_ and Fe_3_O_4_@BTCA materials (Fig. 3), Fourier transform infrared spectroscopy (FT-IR), Thermo-gravimetric analysis (TGA) and Powder X-ray diffraction (PXRD), and for structural confirmation, BET surface area analysis, Scanning electron microscopy (SEM) and Transmission electron microscopy (TEM) analyses were performed.

The FT-IR spectra of Fe_3_O_4_ and Fe_3_O_4_@BTCA materials are shown in Fig. 3A,B. The characteristic absorption bands for Fe_3_O_4_ material appear at 585, 635, 1405, 1625, and 3426 cm^−1^, whereas, for Fe_3_O_4_@BTCA material, bands appear at 585, 635, 1385, 1491, 1581, 1625, 3426 cm^−1^. The common peaks at 585 and 635 cm^−1^ can be assigned to Fe-O bond vibrations^52^, while broad absorption bands appearing at 1625, 1405, and 3426 cm^−1^ arise due to O-H bond vibration of water molecules^53–55^ adsorbed on the surface of both materials. The appearance of additional peaks at 1385, 1491 and 1581 cm^−1^ in the case of Fe_3_O_4_@BTCA indicates the presence of BTCA group. Among these new peaks, the peak at 1581 cm^−1^ can be attributed to asymmetric C-O stretching of carboxylate groups, while the peaks at 1491 and 1485 cm^−1^ are due to aromatic C = C bond vibrations of benzene and the symmetric stretching of carboxylate groups, respectively^56^. Further to ensure the functionalization of BTCA on to the Fe_3_O_4_ surface, UV-Vis spectrum of aqueous BTCA solution was compared with that of aqueous Fe_3_O_4_@BTCA suspension (Fig. 3C). The absorption peak at 220 nm of BTCA was found shifted to 211 nm in Fe_3_O_4_@BTCA, indicating presence of BTCA on the Fe_3_O_4_ surface in deprotonated form^57^.

The TGA curves for Fe_3_O_4_ and Fe_3_O_4_@BTCA are depicted in Fig. 3D. Both materials show an initial small weight loss at temperatures below 200 °C due to desorption of adsorbed water molecules onto their surfaces. The higher weight percentage loss in the case of Fe_3_O_4_@BTCA (2.50%) compared to Fe_3_O_4_ (1.07%) indicates more water adsorbed on Fe_3_O_4_@BTCA due to stronger hydrogen bonding facilitated by the presence of free carboxylate groups. The second region showing 7.01% weight loss in Fe_3_O_4_@BTCA compared to 1.51% in Fe_3_O_4_ indicates the presence of higher organic contents (BTCA) in the case of former. As expected, both materials show similar thermal decomposition behavior beyond 500 °C. Overall, a comparison of three decomposition segments in both materials hints a successful grafting of BTCA group on to the surface of Fe_3_O_4_. Presence of BTCA group on to the surface of Fe_3_O_4_ in Fe_3_O_4_@BTCA is also supported by its EDX spectrum, where peak corresponding to carbon can be clearly seen (Figure S1 in ESI).

The structural information was verified by the powder X-ray diffraction (PXRD) technique. The powder X-ray diffraction patterns of both materials are illustrated in Fig. 3E. Both materials have six characteristic peaks at 2θ = 30.18, 35.49, 43.30, 53.77, 57.21, and 62.83 which correspond to the (220), (311), (400), (422), (511), and (440) reflection planes, respectively, of an fcc magnetite. The crystalline structure of these materials can be established with the standard pattern of Fe_3_O_4_ (ICDD card No 19-0629), which supports that both materials have a pure Fe_3_O_4_ phase with a spinel structure. As evident from the diffraction pattern, immobilization of the BTCA group does not change the Fe_3_O_4_ phase. Using Scherrer equation on PXRD line broadening, the calculated average particle size of Fe_3_O_4_ is found to be 12 nm. The (311) plane mean diffraction peak at 2θ = 35.49 was used to determine the particle size because of its intense nature.

**Figure 4:**
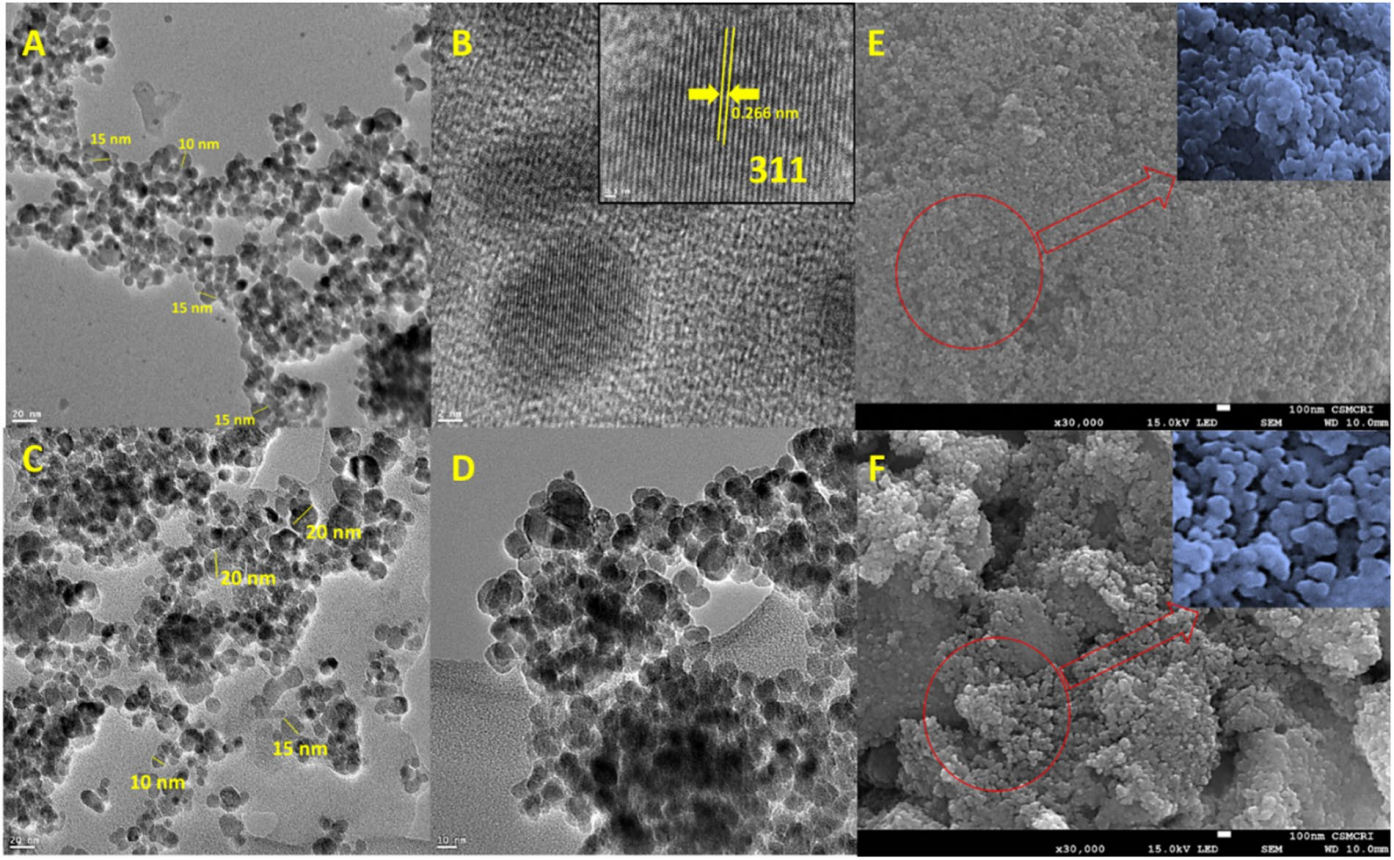
TEM images of (**A**) Fe_3_O_4_, (**B**) Fe_3_O_4_ (HRTEM), (**C**) Fe_3_O_4_@BTCA, (**D**) Fe_3_O_4_@BTCA@C.R. SEM images of (**E)** Fe_3_O_4_ and (**F)** Fe_3_O_4_@BTCA.

TEM images of as-synthesized Fe_3_O_4_, Fe_3_O_4_@BTCA, and Congo Red dye loaded species (Fe_3_O_4_@BTCA@C.R) are shown in Fig. 4A–D. As observed from the TEM images, the spherical shape of the particles is quite evident for all three materials, which is also observed in the SEM images of Fe_3_O_4_ and Fe_3_O_4_@BTCA (Fig. 4E,F). The estimated mean diameter for the Fe_3_O_4_ particle is 10–15 nm (Fig. 4A), which compares well with that obtained from PXRD, and increases to 20 nm after functionalization with BTCA (Fig. 4C). In the HRTEM of Fe_3_O_4_, the 311 planes of spherical Fe_3_O_4_ can be clearly seen with an inter-planar distance of 0.266 nm (Fig. 4B).

The porous structures of as-synthesized Fe_3_O_4_ and Fe_3_O_4_@BTCA were analyzed by N_2_ adsorption-desorption isotherm using a multipoint BET method within the relative pressure (P/Po) range of 0.05–1 (Figure S2A), and by BJH pore size distribution analysis (Figure S2B). The pore sizes, pore volumes, and BET surface areas of both materials are tabulated in Table S1 in ESI. Both materials exhibit type IV isotherms with H3hysteresis loops at relatively high pressure region P/Po = 0.8–1. BJH pore size distribution shows that the surface functionalization by BTCA does not alter any appreciable change in pore size of Fe_3_O_4_@BTCA (12.31 nm) compared to that of Fe_3_O_4_ (12.41 nm). This indicates that the immobilization of BTCA occurs on the outer surface of Fe_3_O_4_. The enhanced surface area of Fe_3_O_4_@BTCA (72.35 m^2^/g) compared to that of Fe_3_O_4_ (46.66 m^2^/g) can be attributed to a higher dispersity of Fe_3_O_4_@BTCA NPs due to negatively charged surface repulsion.

Surface charge analyses by zeta-potential calculations of Fe_3_O_4_ and Fe_3_O_4_@BTCA show that the former, having −7.21 mV potential, possess significantly lesser negative surface charge than the later having −17.8 mV. Observation of the higher negative surface charge in the case of Fe_3_O_4_@BTCA is understandable as it bears BTCA groups with a number of free carboxylate groups covering the surface. For Fe_3_O_4_@BTCA, information about the higher surface area from BET and the idea of greater negative surface charge from surface charge analysis together support our assumption of obtaining better adsorption efficiency by immobilization of BTCA groups.

As the surface of Fe_3_O_4_@BTCA bears free carboxylate groups, reprotonation and de-protonation are expected to affect its surface charge. Therefore, the surface charge analysis of Fe_3_O_4_@BTCA was carried out at various pH, and the calculated zeta-potentials at different pH are shown in Fig. S3 in ESI. Due to excess protonation, the calculated zeta-potential is in the positive range till pH 5, whereas, an increasing trend of the negative surface is observed upon increasing pH above 5 because of deprotonation of –COOH groups on the surface. This observation supports our proposed mechanism of dye adsorption onto the Fe_3_O_4_@BTCA surface, as discussed later.

**Figure 5:**
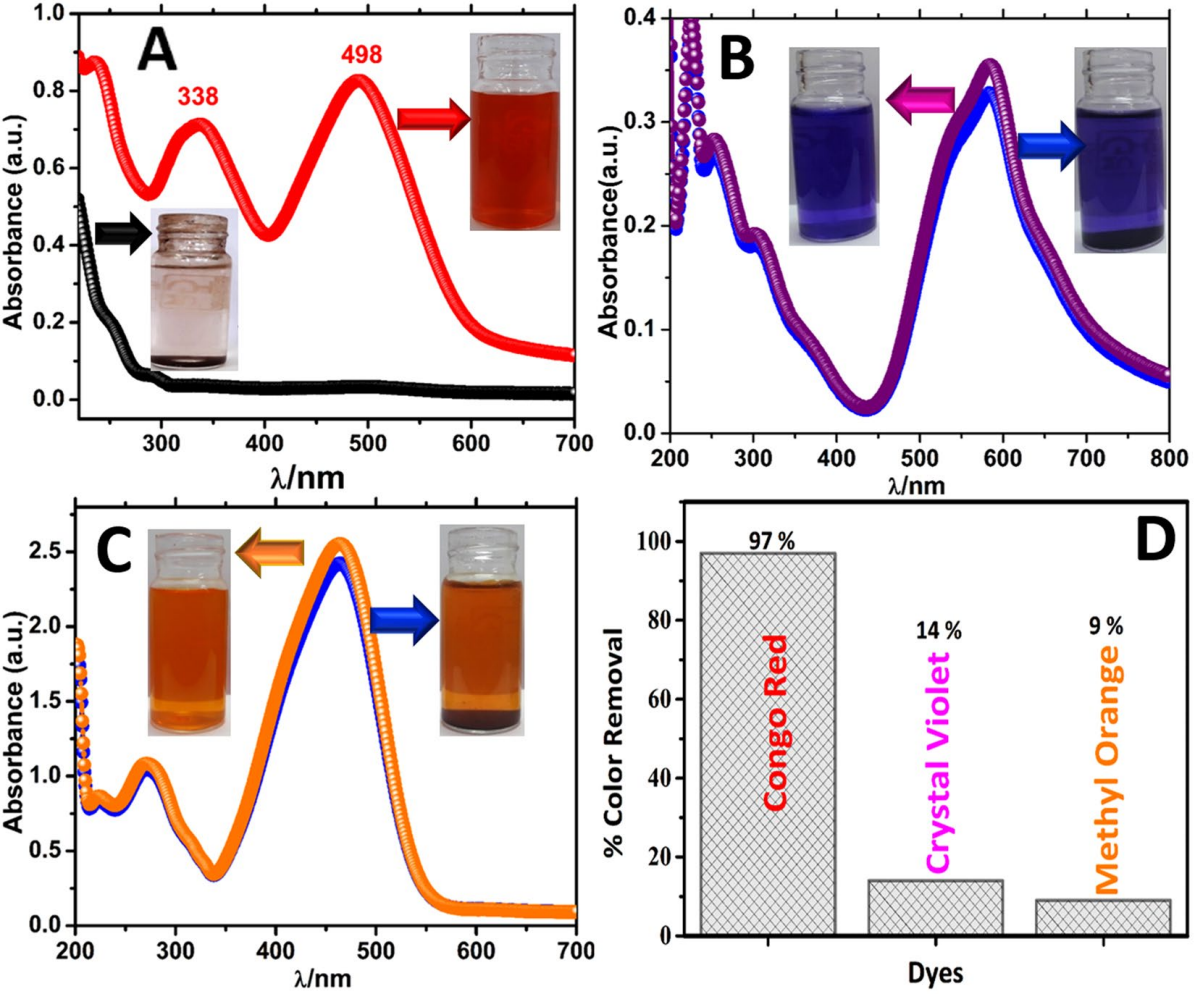
UV-Vis spectra of (**A)** C.R dye, (**B)** C.V dye, (**C)** M.O dye aqueous solutions before and after shaking with Fe_3_O_4_@BTCA, (**D)** Bar diagram for percentage removal of C.R, C.V, and M.O dyes (percentage color removal = 100 × (C_i_-C_f_.)/C_i_, C_i_ and C_f_ are concentrations of dyes before and after shaking with Fe_3_O_4_@BTCA, respectively).

The ability of Fe_3_O_4_@BTCA to adsorb dyes on to its surface, leading to the color removal of aqueous dye solutions was investigated towards C.R (Congo red), M.O (Methyl Orange), and C.V (Crystal Violet) by UV-Vis absorption spectroscopy. Figure 5 shows the color removal of aqueous dye solutions of C.R, M.O, and C.V before and after shaking the solution with Fe_3_O_4_@BTCA for 15 minutes. It is evident that Fe_3_O_4_@BTCA does selective adsorption of C.R dye leading to 97% color removal (Fig. 5A). However, in the case of M.O and C.V, only 14% and 9% color removal, respectively, were observed under similar adsorption conditions, suggesting no considerable absorption of M.O and C.V (Fig. 5B,C). UV-Vis spectra also indicate that the two intense absorption peaks of C.R at 338 and 498 nm vanish entirely after treatment with Fe_3_O_4_@BTCA. However, a similar phenomenon for M.O and C.V was not observed as the intensities of their UV-Vis absorption peaks remain almost unaltered after treatment with Fe_3_O_4_@BTCA. We also investigated similar color removal studies for C.R by as-synthesized Fe_3_O_4_ NPs, which indicate only 12% color removal (Fig. S4 in ESI). To reach an optimum condition for C.R dye adsorption, its color removal studies were performed at different dye concentrations (20–100 ppm), Fe_3_O_4_@BTCA doses (5 and 15 mg), and pH conditions (2–10). The resulting graph and bar diagram with corresponding values are given in Fig. S5 in ESI. An increasing percentage color removal trend was observed upon increasing the pH of the solution until pH 7 (97% color removal), which remains constant after further increase in pH. Best adsorption in terms of percentage color removal (97%) was observed with 20 mg catalyst dose in 20 ppm solution of C.R dye.

Further to support the fact that the color removal of Congo red dye is caused by its adsorption on to the surface of Fe_3_O_4_@BTCA, using FT-IR and UV-Vis spectroscopy, we also ensured the formation of Fe_3_O_4_@BTCA@C.R species containing adsorbed C.R dye on the surface of Fe_3_O_4_@BTCA. Fe_3_O_4_@BTCA@C.R was obtained by stirring 50 mg of Fe_3_O_4_@BTCA in 100 ppm aqueous solution of C.R dye for 15 minutes, followed by its magnetic separation, washing and drying at 70 °C for 24 h. The UV-Vis absorption spectrum of this material shows two weak peaks at 498 and 338 nm (Fig. S6 in ESI), indicating the presence of C.R dye on the surface of Fe_3_O_4_@BTCA material. The same is also supported by FT-IR spectrum (Fig. S7 in ESI), in which appearance of a peak at 1047 cm^−1^ is due to vibration of S=O group present in C.R dye^28^.

In order to estimate the adsorption capacity of Fe_3_O_4_@BTCA towards C.R dye and to understand the adsorption behavior (Langmuir type or Freundlich type), adsorption isotherm studies were performed through batch experiments. Prior to the adsorption study, a calibration plot (Fig. S8 in ESI) of concentration versus absorbance was drawn by recording UV-Vis spectra of aqueous C.R dye solutions of different concentrations. Adsorption study was performed by allowing adsorption of 10 batches of 50 mL aqueous C.R dye solutions of varying concentrations (10–100 ppm) on a fixed amount of Fe_3_O_4_@BTCA material (5 mg) through vigorous shaking for 15 minutes. After completion of adsorption, the equilibrium concentration of C.R dye in each magnetically separated filtrate was calculated by comparing its UV-Vis spectrum with the calibration plot. The equilibrium adsorption capacity (q_e_) for each batch adsorption can be calculated using Eq. 1 ^58–61^.

1 $${q}_{e}=({C}_{i}-\,{C}_{e})V/W$$

**Figure 6:**
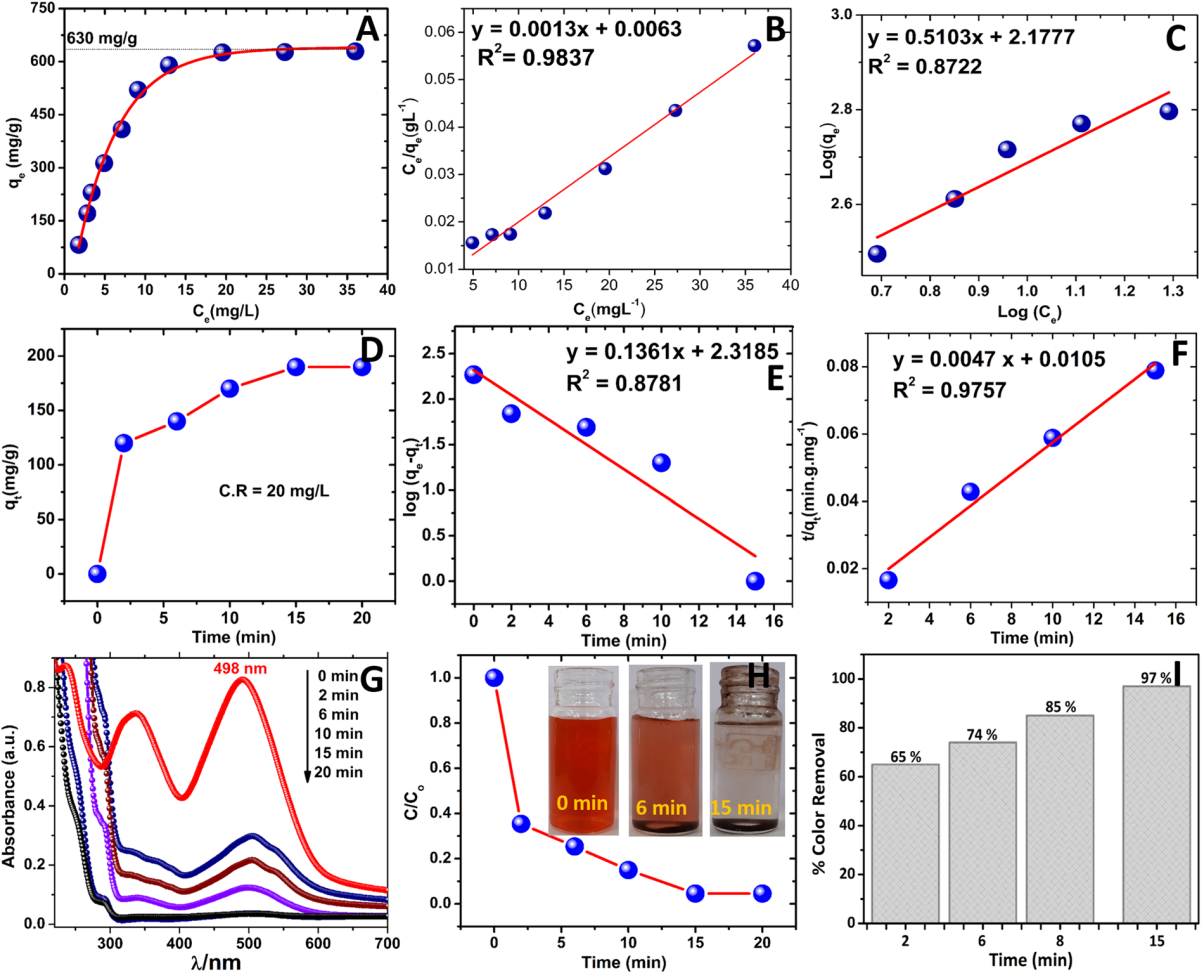
**(A)** Experimental maximum adsorption isotherm of C.R by Fe_3_O_4_@BTCA, (**B)** Langmuir adsorption isotherm, (**C)** Freundlich adsorption isotherm of C.R dye on Fe_3_O_4_@BTCA, (**D)** Experimental adsorption capacity with time, (**E)** Pseudo-first-order, (**F)** Pseudo-second-order kinetic plot, (**G)** UV-Vis spectra of C.R dye with increasing time, (**H)** Kinetic plot of C.R dye, (**I)** Percentage color removal of C.R dye with time.

Where *C*_*i*_ and *C*_*e*_ are initial and equilibrium concentrations (mg/L) of C.R dye, respectively. *V* is the volume of C.R solution (50 × 10^−3^L), *W* is the amount of Fe_3_O_4_@BTCA (5 × 10^−3^g). The adsorption capacity of Fe_3_O_4_@BTCA for C.R dye can be estimated from the plot of *q*_*e*_ and *C*_*e*_ values obtained at a different concentration of C.R dye (Fig. 6A). From Fig. 6A, the adsorption capacity of our material towards C.R dye was found to be 630 mg/g, which is significantly higher than the earlier reported Fe_3_O_4_ based sorbents listed in Table S3 in ESI^38–40,45–47,62–64^.

The significantly higher adsorption capacity value for Fe_3_O_4_@BTCA supports our initial expectations of achieving high adsorption capacity through immobilization of BTCA on the Fe_3_O_4_ surface by enhancing the surface area and making strong H-binding sites available to promote the dye adsorption.

Adsorption behavior of C.R dye on the surface of Fe_3_O_4_@BTCA was studied by comparing the fitting of adsorption parameters in the Langmuir and Freundlich type adsorption isotherm equations. Homogeneous monolayer adsorption follows the Langmuir adsorption isotherm given in Eq. 2 ^63^.

2 $$\frac{{C}_{e}}{{q}_{e}}(Langmuir)=\frac{{C}_{e}}{{q}_{max}}+\frac{1}{{q}_{max}\times {K}_{L}}$$

Where *C*_*e*_ and *q*_*e*_ represent the same parameters as mentioned before. *q*_*max*_ is adsorption capacity (630 mg/g), and *K*_*L*_ (L/mg) is Langmuir equilibrium constant, which is related to adsorption energy.

The possibility of multilayer adsorption is allowed in the Freundlich adsorption model, and its adsorption isotherm is given in Eq. 3 ^64^.

3 $$log{q}_{e}(Freundlich)=log{K}_{f}+\frac{1}{n\,log\,{C}_{e}}$$

Where *K*_*f*_ and *n* are Freundlich constants related to adsorption capacity and adsorption intensity, respectively.

From Fig. 6B,C, showing Langmuir and Freundlich isotherms, respectively, it is clear that the experimental adsorption isotherm is best fitting with monolayer Langmuir adsorption isotherm (R^2^ = 0.9837 for Langmuir and 0.8722 for Freundlich). Other parameters (K_L_, q_max_, K_f_, and n) for both adsorption isotherms are given in Table S2A.

In order to understand the adsorption kinetics, the order of the adsorption process was ascertained by fitting the experimental data into the pseudo-first-order and pseudo-second-order equations (Eqs. 4 and 5)^65^.

4 $$\log ({q}_{e}-{q}_{t})=\,\log \,{q}_{e}-\frac{{K}_{1}}{2.303\,}\times t$$

5 $$\frac{t}{{q}_{t}}=\frac{1}{{K}_{2}\times {q}_{e}^{2\,}}+\,\frac{t}{{q}_{e}}$$

The equilibrium adsorption capacity (*q*_*t*_) towards C.R dye was calculated by UV-Vis spectroscopy at different time intervals (*t*) taking 20 ppm initial aqueous solution of C.R dye with 5 mg Fe_3_O_4_@BTCA. With increasing time (*t* = 2, 6, 8, 15 and 20 min), the obtained values of *q*_*t*_ are plotted in Fig. 6D. The final value of equilibrium adsorption capacity *q*_*e*_ (192 mg/g) is achieved in 15 min and remains constant with further increase in time interval. Upon fitting the value of q_e_ (192 mg/g) and that of q_t_ obtained at a different time interval in straight line Eqs. 4 and 5 (Fig. 6E,F), the best fitting (R^2^ = 0.9757) was obtained for pseudo-second-order adsorption (remaining kinetic parameters are listed in Table S2B). The extent of color removal with time was also studied under same adsorption conditions. The change in UV-Vis spectra of C.R dye and corresponding percentage change in color upon stirring with Fe_3_O_4_@BTCA for the different time intervals is given in Fig. 6G. The steep nature of *C*_*o*_/*C* vs. time plot (Fig. 6H) suggests rapid dye adsorption up to 2 minutes, which subsequently starts flattening indicating a decrease in the rate of dye adsorption. The bar diagram in Fig. 6I illustrates that within 2 minutes 65% of color removal can be achieved, and further stirring till 15 minutes leads to 97% dye color removal.

The process of adsorption is facilitated by various factors including, high surface area, high surface charge, and the presence of compatible functional groups. As evident from results obtained from BET study and zeta potential calculation, surface immobilization by BTCA group on to the Fe_3_O_4_ NPs led to a significant increase in both surface area and surface charge. Besides these, under synthetic conditions (pH 9–10), the presence of BTCA groups on the Fe_3_O_4_ surface provides lots of free carboxylate groups (–COO^−^) covering the surface of Fe_3_O_4_. During interaction of Fe_3_O_4_@BTCA and C.R dye, the free carboxylate groups facilitate the adsorption process through formation of a strong H-bond with free –NH_2_ groups of C.R dye as depicted in Fig. 1, leading to the formation of Fe_3_O_4_@BTCA@C.R. The involvement of H-bond in the formation of Fe_3_O_4_@BTCA@C.R is supported by the fact that the asymmetric C-O stretching of carboxylate groups in Fe_3_O_4_@BTCA at 1581 cm^−1^ almost disappears in Fe_3_O_4_@BTCA@C.R (Fig. S7 in ESI).

**Figure 7:**
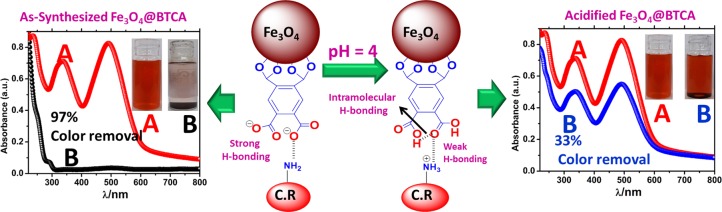
Plausible adsorption mechanism of C.R dye onto Fe_3_O_4_@BTCA surface.

As strong H-bonding plays a crucial role in the adsorption of C.R dye on Fe_3_O_4_@BTCA, adsorption capacity should drastically decrease in the absence of a strong H-bonding. To confirm this, the free carboxylate groups (-COO^−^) on Fe_3_O_4_@BTCA were re-protonated to convert them all to corresponding carboxylic acid (-COOH), whose H-bond acceptor ability is much less than the carboxylate groups. When acidified Fe_3_O_4_@BTCA was subjected to C.R dye adsorption under similar conditions, the observed percentage of color removal was 33% only (Fig. 7). This is also supported by the calculation of zeta potential at various pH (Fig. S3 in ESI), which suggests that in acidic conditions (till pH 5), material bears a positive surface charge. The observation of 33% color removal can be attributed to the fact that under acidic conditions, free –NH_2_ of the dye will also be converted to -NH_3_^+^ having stronger H-bond donor character than –NH_2_.

The observation of significantly lowered adsorption capacity of acidified Fe_3_O_4_@BTCA for C.R dye also corroborates that involvement of H-bonding, owing to the presence of BTCA groups on the Fe_3_O_4_ surface, is an important factor responsible for unexpectedly high adsorption capacity of Fe_3_O_4_@BTCA material. In the case of Methyl orange and Crystal violet, however, such H-bonding is not possible because of the unavailability of amine hydrogen atoms due to the presence of tertiary amines in both dyes. Therefore, no appreciable color change is observed when M.O and C.R dye solutions are shaken with Fe_3_O_4_@BTCA (Fig. S9 in ESI).

**Figure 8:**
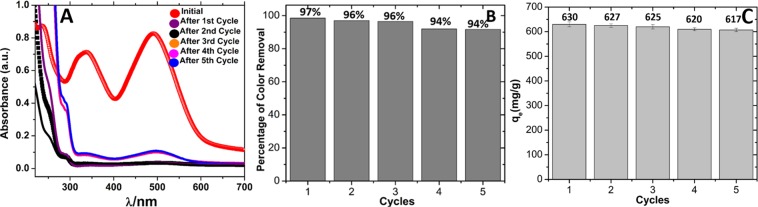
(**A)** UV-Vis Spectra of C.R dye solution after several cycles, (**B)** Percentage of color removal of C.R dye after several cycles, (**C)** Adsorption capacity of Fe_3_O_4_@BTCA material in five cycles.

In order to perform efficient pollutant sequestration through adsorption, the sorbent should have the ability to be recycled without undergoing any considerable reduction in its adsorption capacity. To investigate such features in our system, the reusability test of Fe_3_O_4_@BTCA was studied for five cycles by adsorption/desorption and regeneration. The adsorption study was performed by recording the UV-Vis spectra of the remaining filtrate after completion of adsorption in each cycle. The UV-Vis spectra recorded for each cycle is shown in Fig. 8A, and the corresponding percentage color removal ability is represented by a bar diagram in Fig. 8B. From Fig. 8A,B, it is clear that no appreciable reduction (only ~3%) in percentage color removal ability Fe_3_O_4_@BTCA was observed in five cycles. We also calculated the change in adsorption capacity of our material by performing a batch experiment for each cycle. The values of adsorption capacity for each cycle are given in Fig. 8C as a bar diagram, which shows that, in five cycles, the adsorption capacity of our material is reduced by 2% only. Separation of C.R dye loaded Fe_3_O_4_@BTCA from aqueous dye solution by magnet is shown in Fig. S10 in ESI.

## Conclusion

A new Fe_3_O_4_@BTCA material has been synthesized by surface functionalization of Fe_3_O_4_ NPs with 1,2,4,5-Benzenetetracarboxylic acid for rapid and selective adsorption of Congo red dye in an aqueous medium. Adsorption studies show that the Fe_3_O_4_@BTCA material exhibits high adsorption capacity for C.R dye (630 mg/g) and can be reused for multiple adsorption-desorption cycles without undergoing any considerable loss in its performance. Adsorption isotherm and kinetics studies reveal that the adsorption of C.R dye on the Fe_3_O_4_@BTCA surface takes place by the Langmuir adsorption model and follows pseudo-second-order adsorption kinetics.

The high adsorption capacity, selectivity and better recyclability of the material can be attributed to surface functionalization ligand BTCA, which, owing to its four uniquely positioned carboxylate group, offers strong surface encourage and participates in the formation of a strong H-bonding only with Congo red dye, containing a primary amine group. This report opens up new possibilities for 1,2,4,5-Benzenetetracarboxylic acid to be explored for surface functionalization of other materials to achieve improved targeted applications.

## Experimental Section

### Materials

All reagents and chemical used were of analytical standards. In all experiments, double distilled-deionized water was used. FeCl_3_.6H_2_O (98%), FeCl_2_.4H_2_O (99%) and 1,2,4,5-Benzenetetracarboxylic acid (BTCA) were purchased from Sigma Aldrich. Ammonia solution (25%), HCl, Congo Red (C.R), Methyl Orange (M.O), and Crystal Violet (C.V) were purchased from Spectrochem. pH buffer tablets were purchased from SRL.

### Synthesis of Fe_3_O_4_ nanoparticles

Fe_3_O_4_ nanoparticles were synthesized by chemical co-precipitation method^51^. Initially, FeCl_3_.6H_2_O (5.46 g, 20 mmol) and FeCl_2_.4H_2_O (2 g, 10 mmol) were dissolved in 400 mL deionized water and kept at room temperature for 15 min with vigorous stirring under a nitrogen atmosphere. After that, 10 mL of ammonia solution (25%) was added drop by drop into the salt solution, and stirring was continued for another 2 h at 80 °C. The solution was cooled, and solid black particles were magnetically separated and repeatedly washed with Millipore water. The particles were finally dried in a vacuum oven at 70 °C for overnight.

### Direct functionalization of 1,2,4,5-Benzenetetracarboxylic acid (BTCA) onto Fe_3_O_4_ surface

In a direct functionalization procedure, 1 g of Fe_3_O_4_ was taken in a 500 mL round bottom flask containing 200 mL of deionized water. To this suspension, 2 g BTCA (3.905 mmol) and 5 mL aqueous NH_4_OH solution were added until the pH reached 11.0. The suspension was refluxed for 24 h in a nitrogen atmosphere with constant stirring. The resulting Fe_3_O_4_@BTCA material was separated through magnate and washed thoroughly with deionized water thrice to remove unreacted BTCA. The residue was dried under vacuum at 70 °C for 24 h to give brown colored Fe_3_O_4_@BTCA material.

### Instrumentation

Electronic spectra of suspension were recorded on Varian UV−visible spectrophotometer (Carry 100 Bio). FT-IR spectra (4000–400 cm^−1^) were collected on a Perkin-Elmer GX spectrophotometer (U.S.A) using KBr disks. The zeta potentials were recorded using a Malvern instrument (Zetasizer, Nano series, Nano-ZS90). Scanning electron microscope (SEM) (Leo series 1430 VP) equipped with INCA was used to determine the morphologies of samples. Transmission electron microscopy (TEM) was performed using a JEOL JEM 2100 microscope. Thermogravimetric analysis (TGA) was done using Mettler Toledo TGA/DSC 1 analyzer. Powder X-ray diffraction (XRD) measurements were conducted on Rigaku smart lab automated multipurpose X-ray diffractometer system with CuKα1 radiation (λ = 1.540593 Å) in the 2θ range of 20–70° at scanning speed of 3° per minute with 0.01° scan step size. The Brunauer− Emmett−Teller (BET) surface area analysis was conducted on an Autosorb iQ, version 1.11 (Quantachrome Instruments).

### UV-visible studies

UV-Vis spectra of Fe_3_O_4_, Fe_3_O_4_@BTCA, and Fe_3_O_4_@BTCA@Dye were recorded using suspension of 1 mg of material with 3 mL of deionized water in 1 cm quartz cuvette within the range of 200–800 nm. The uniform suspension was obtained by shaking quartz cuvette on to a vertex shaker for five minutes before the recording of each spectrum. UV-Vis spectra of dyes were recorded in their aqueous solution in the same wavelength range.

### Adsorption study of dye on Fe_3_O_4_@BTCA surface

Batch adsorption experiments were carried out in an aqueous dye solution to determine several adsorption parameters. Adsorption capacity (Q_e_) measurements for Congo red dye were done by taking 5 mg of Fe_3_O_4_@BTCA material in ten batches of 50 ml aqueous dye solution of different concentration (10 ppm to 100 ppm). Each batch containing a suspension of 5 mg of Fe_3_O_4_@BTCA in aqueous dye solution was stirred at room temperature for 15 min (pH of dye solutions ~7.4), after which, the concentration of unabsorbed dye was determined from UV-Vis spectrum of the filtrate. All the filtrations were done using a strong magnet. All the adsorption experiments were repeated thrice to remove the experimental error.

### Reusability test of Fe_3_O_4_@BTCA

The reusability experiment of material was done through simple acid-base treatments by UV-visible spectral studies and calculation of adsorption capacity. Initially, adsorption of 10 batches of 50 mL aqueous C.R dye solutions of varying concentrations (10–100 ppm) was allowed on 5 mg Fe_3_O_4_@BTCA and, as discussed in the previous section, filtrate from each batch was subjected to UV-Vis study to determine the amount of adsorbed C.R dye on the Fe_3_O_4_@BTCA surface. The magnetically isolated sorbent of each batch was washed thoroughly with deionized water and treated with few drops of 0.1 N HCl under vigorous shaking for 5 min and again filtered magnetically to remove the adsorbed C.R dye. After further washing with deionized water twice, the sorbent was treated with few drops of 0.1 N NH_4_OH under vigorous shaking. After filtration and proper washing, the regenerated Fe_3_O_4_@BTCA was dried overnight in a vacuum oven at 70 °C for reuse. The dried material of each batch was again subjected to the same dye adsorption-desorption cycles through acid-base treatment four times. Adsorption parameter from each cycle was determined from the plot of adsorption isotherm using Eq. 2 discussed later.

## Supplementary information

Supplementary information.

## Data Availability

The datasets generated and analyzed during the current study are included in this article and its supplementary information file and also it is available from the corresponding author on reasonable request.
