# Supplementary material for: Selective and Recyclable Congo Red Dye Adsorption by Spherical Fe3O4 Nanoparticles Functionalized with 1,2,4,5-Benzenetetracarboxylic Acid
Source: Sci Rep. 2020 Jan 10;10:111. doi: 10.1038/s41598-019-57017-2 (PMC6954200; doi:10.1038/s41598-019-57017-2)
Supplement: Supplementary file 1 — Supplementary information. [file 41598_2019_57017_MOESM1_ESM.docx]

Supporting Information

**Selective and Recyclable Congo Red Dye Adsorption by Spherical Fe_3_O_4_**

**Nanoparticles Functionalized with 1,2,4,5-Benzenetetracarboxylic Acid**

Sobhan Chatterjee^§^, Nikita Guha^†^, Sarathkumar Krishnan^†^, Amrendra K. Singh^§^, Pradeep Mathur^§^, Dhirendra K. Rai^†^*

^†^ Discipline of Metallurgy Engineering and Materials Science, Indian Institute of Technology Indore, Simrol, Indore 453552, India

^§^ Discipline of Chemistry, Indian Institute of Technology Indore, Simrol, Indore 453552, India

**List of Content**

**Figure S1.** EDX Spectra of Fe_3_O_4_@BTCA showing peak for carbon of BTCA group

**Figure S2.** BET Surface area and BJH Pore size distribution of Fe_3_O_4_ and Fe_3_O_4_@BTCA

**Figure S3.** Surface zeta potential variation of Fe_3_O_4_@BTCA with the change in pH

**Figure S4.** UV-Visible spectra of Congo red dye before and after shaking with Fe_3_O_4_

**Figure S5.** Percentage color removal of C.R dye at various pH, dye concentration and catalyst load

**Figure S6.** UV-Vis spectra of C.R dye loaded Fe_3_O_4_@BTCA (Fe_3_O_4_@BTCA@C.R)

**Figure S7.** FT-IR spectra of C.R dye loaded Fe_3_O_4_@BTCA (Fe_3_O_4_@BTCA@C.R)

**Figure S8.** Calibration plot of concentration of C.R dye versus UV-Vis absorbance

**Figure S9.** The probable explanation for selective adsorption of C.R dye onto Fe_3_O_4_@BTCA surface among C.R, C.V, and M.O dyes.

**Figure S10.** Magnetic separation of C.R loaded Fe_3_O_4_@BTCA material from the aqueous solution of C.R dye.

**Figure S11.** Molecular structures of Congo Red, Crystal Violet, and Methyl Orange dyes.

**Table S1.** Pore size, pore volume and surface area of Fe_3_O4 and Fe_3_O_4_@BTCA.

**Table S2.** **A)** Fitting results of adsorption isotherm data with the Langmuir and Freundlich model for C.R dye, **B)** First and second order Kinetic Parameters for the adsorption of CR on Fe_3_O_4_@BTCA surface

**Table S3.** Comparison of Adsorption Capacities towards C.R on various Fe_3_O_4_ based NPs (Ref. in main manuscript).


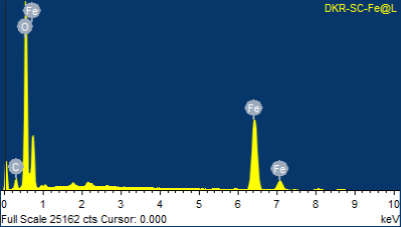


**Figure S1.** EDX Spectra of Fe_3_O_4_@BTCA showing a peak for carbon of BTCA group


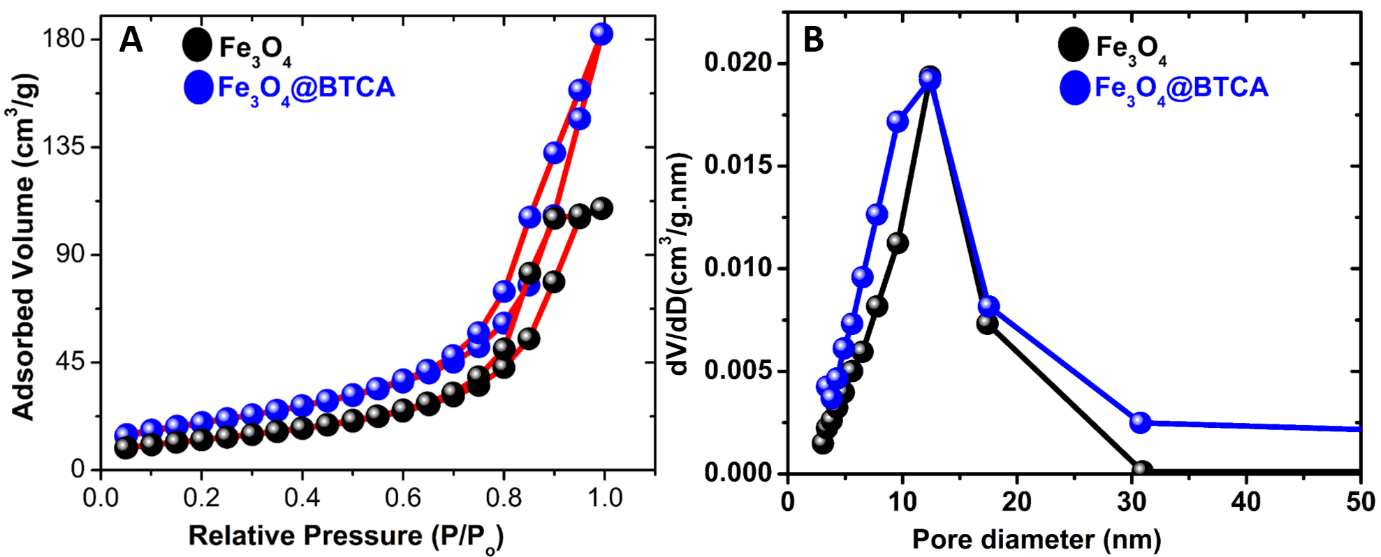


**Figure S2.** BET Surface area and BJH Pore size distribution of Fe_3_O_4_ and Fe_3_O_4_@BTCA


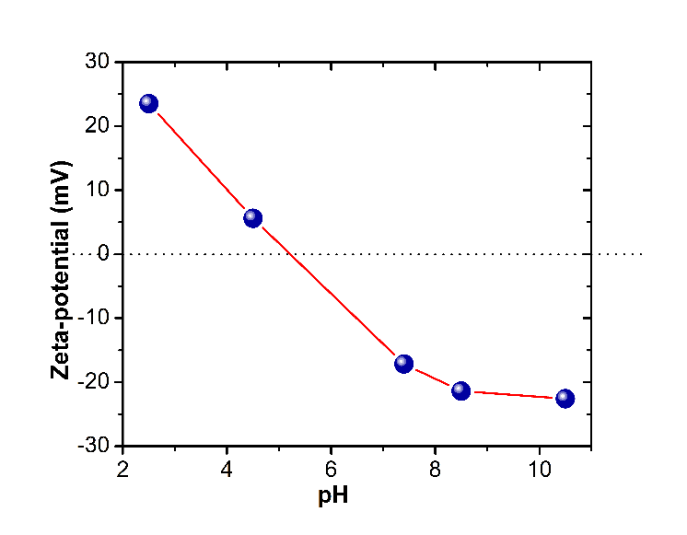


**Figure S3.** Surface zeta potential variation of Fe_3_O_4_@BTCA with the change in pH of the suspension


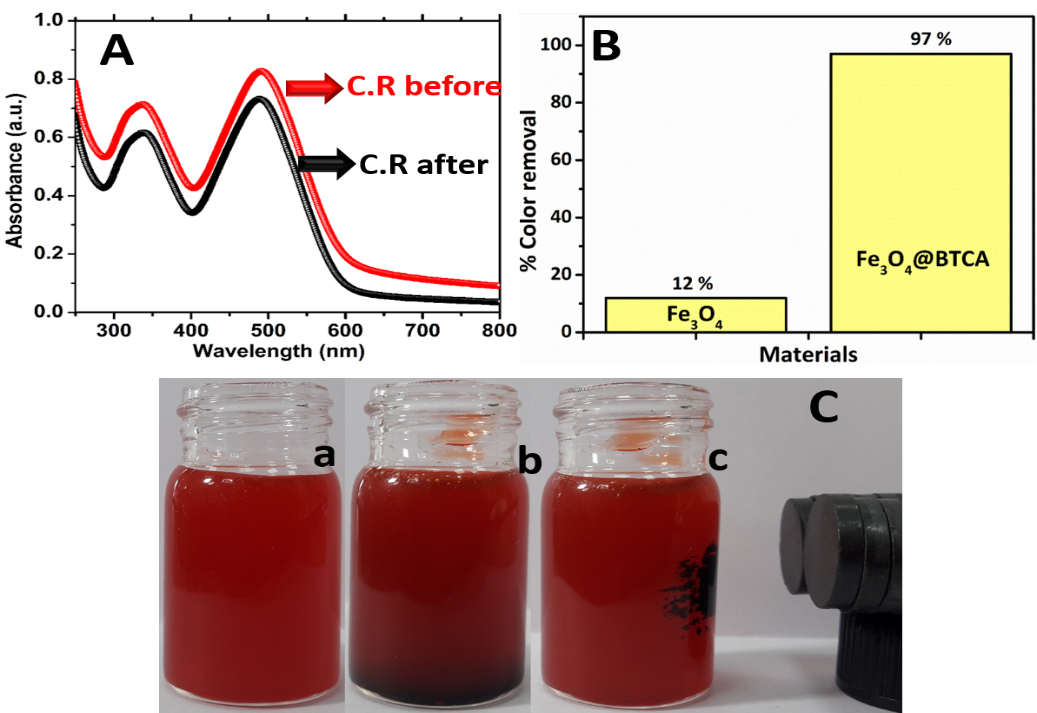


**Figure S4.** **A**) UV-Vis spectra of C.R dye solution before and after shaking with as-synthesized Fe_3_O_4_ nanoparticles, **B**) % color removal of C.R dye by Fe_3_O_4_ (12%) and Fe_3_O_4_@BTCA (97%), **C**) Bottle **a** containing untreated congo red dye solution, Bottle **b** containing C.R dye solution shaken with of Fe_3_O_4_ for 15 min, Bottle **c** showing application of external magnet on bottle **b**.


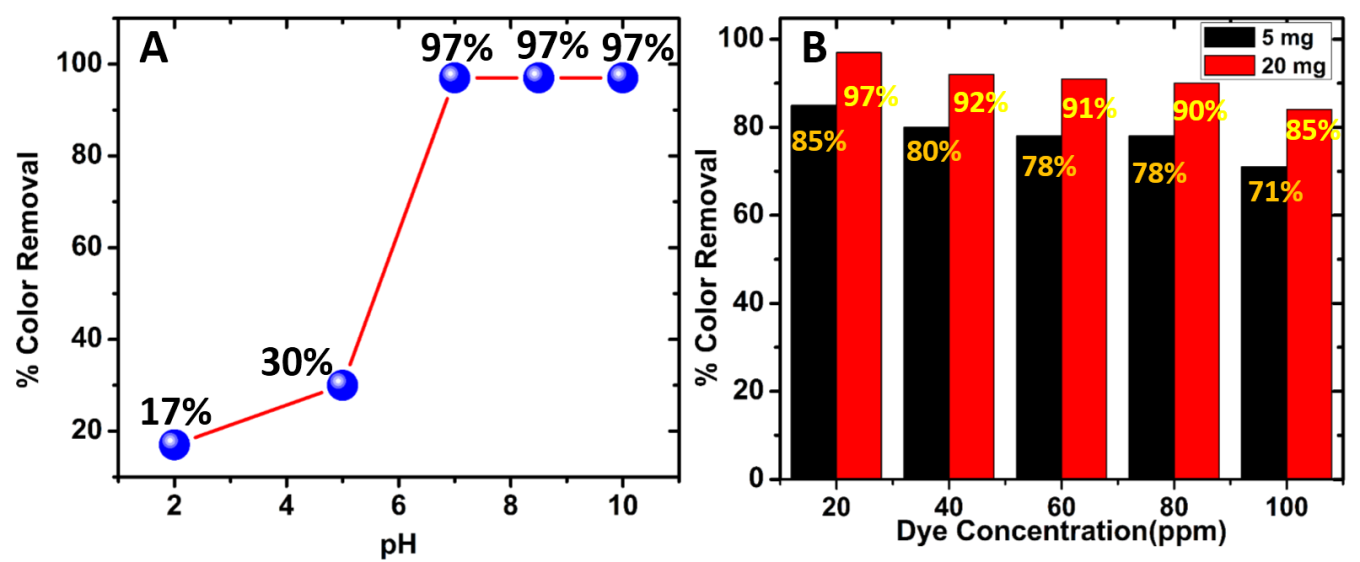


**Figure S5.** **A**) Color removal graph of C.R dye at various pH by Fe_3_O_4_@BTCA, **B**) Color removal of C.R dye at various concentrations of dye solution and two different doses of Fe_3_O_4_@BTCA.


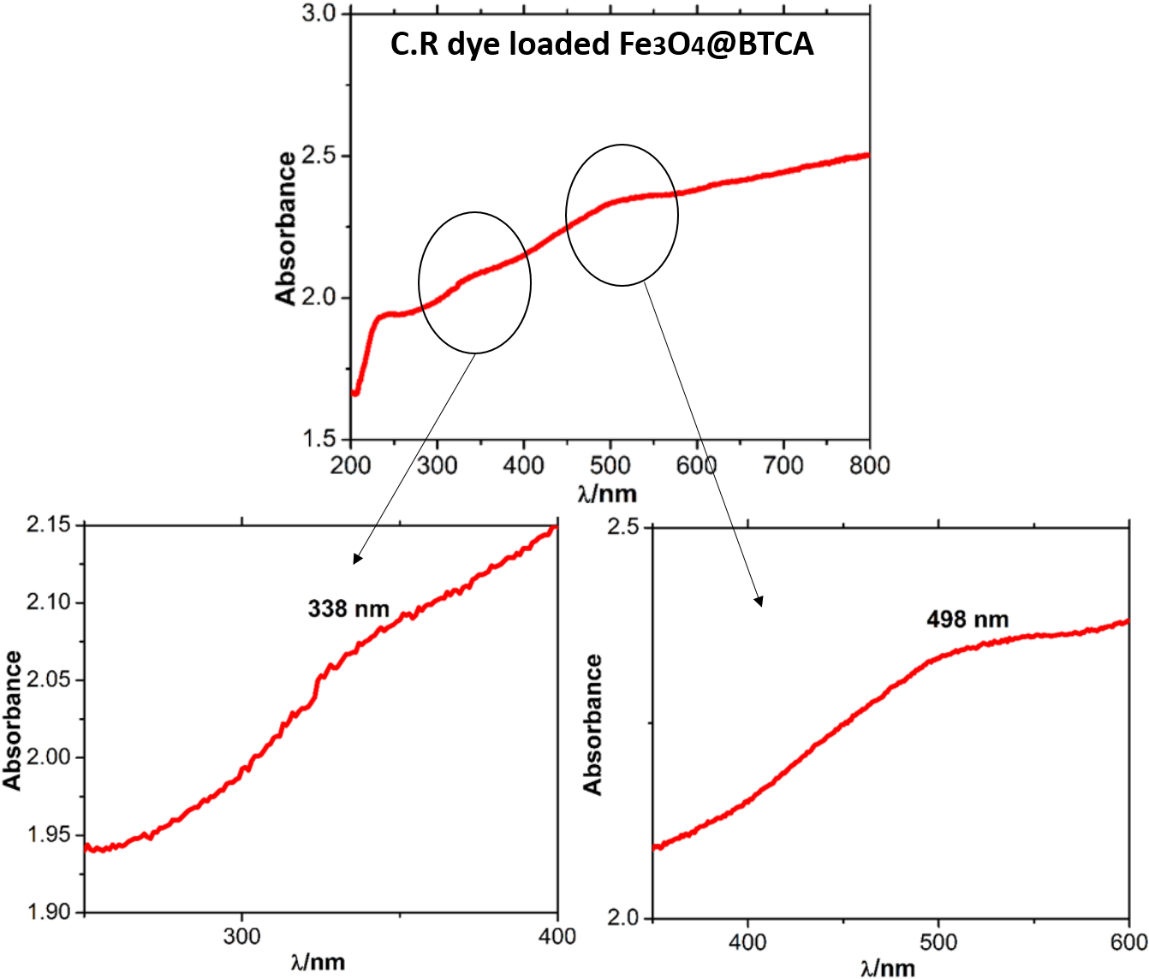


**Figure S6**. UV-Vis spectra of CR dye loaded Fe_3_O_4_@BTCA (Fe_3_O_4_@BTCA@C.R)


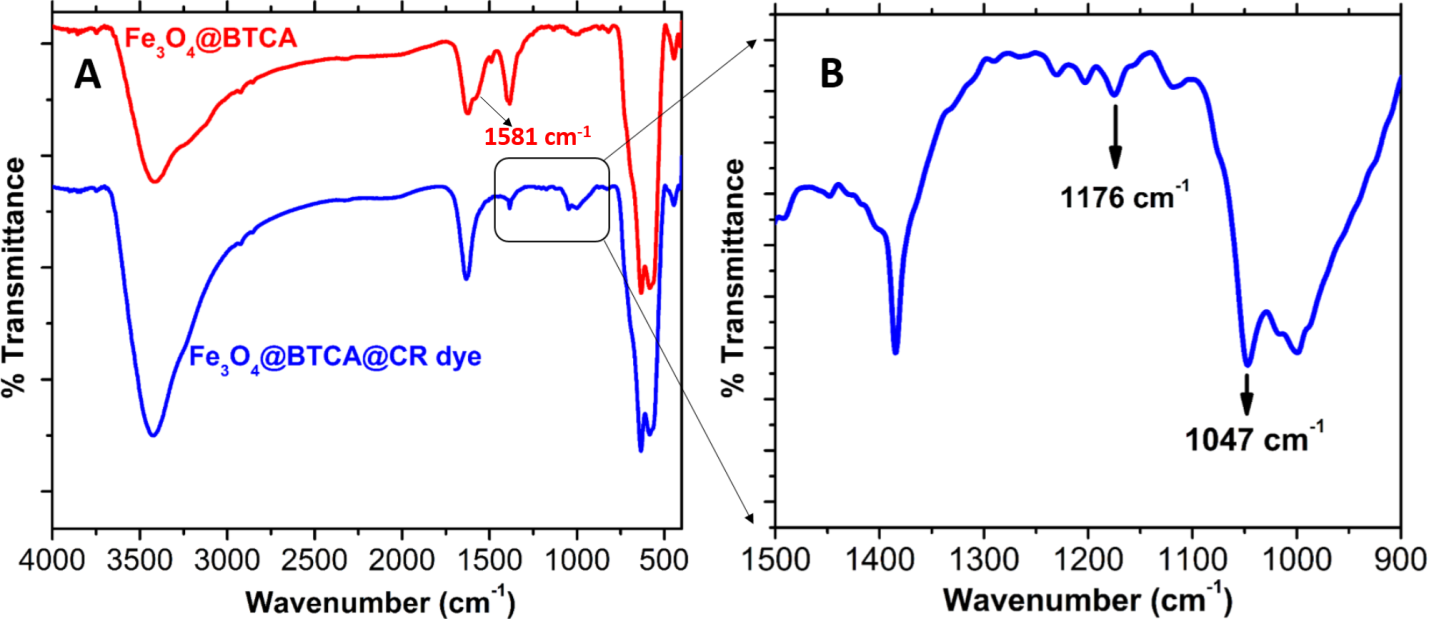


**Figure S7.** FT-IR spectra of CR dye loaded Fe_3_O_4_@BTCA (Fe_3_O_4_@BTCA@CR)


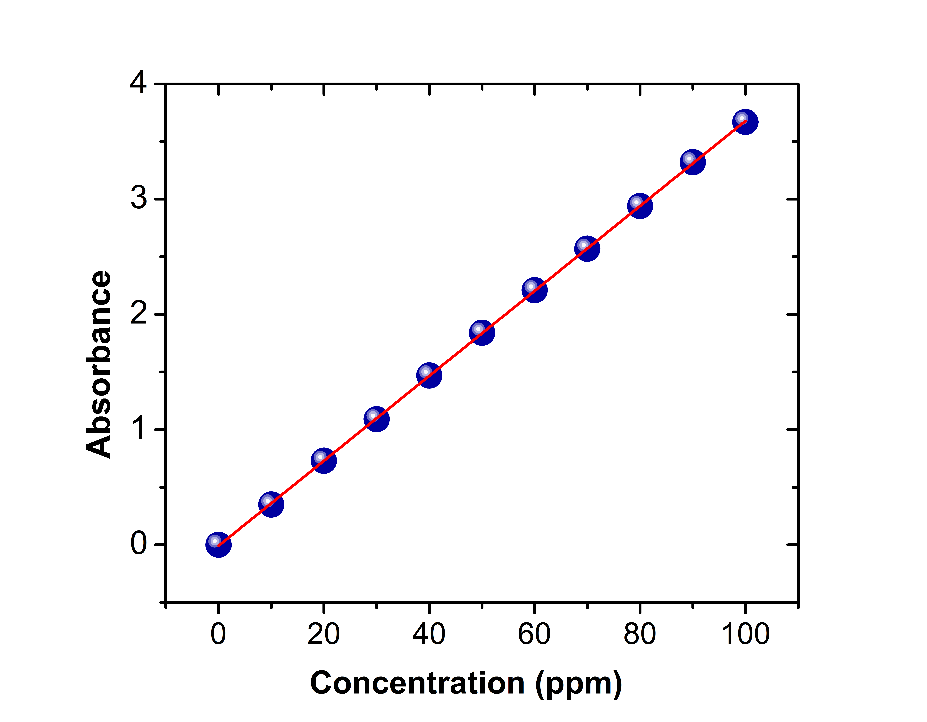


**Figure S8.** Calibration plot of concentration of C.R dye versus UV-Vis absorbance


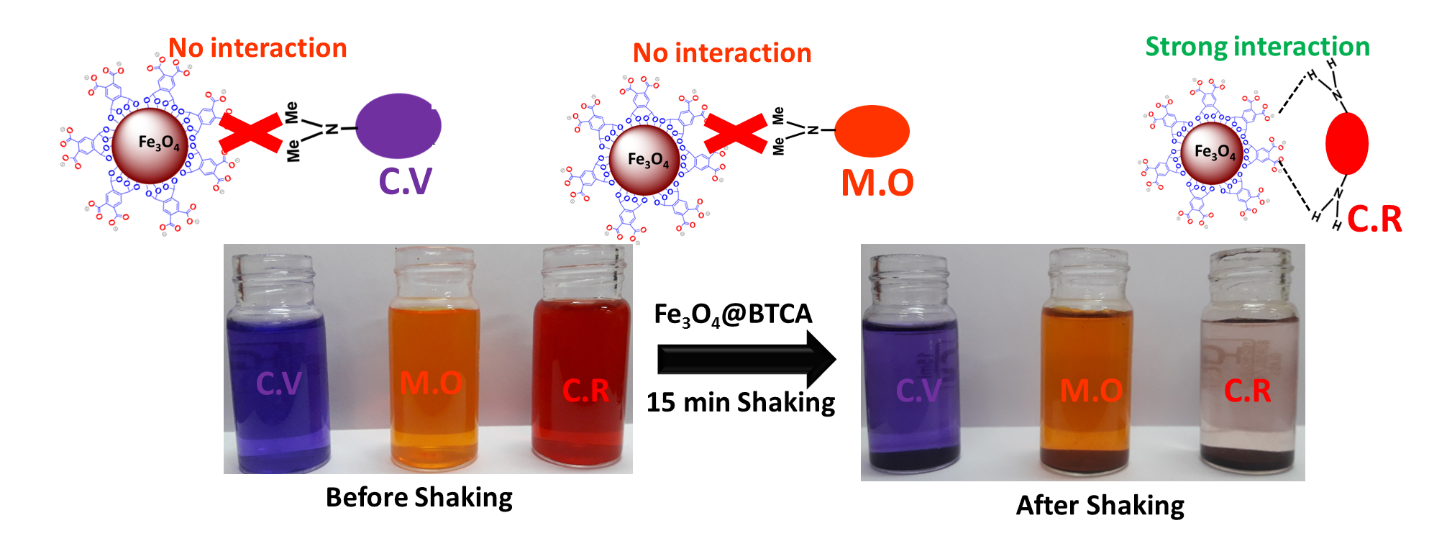


**Figure S9.** The probable explanation for selective adsorption of C.R dye onto Fe_3_O_4_@BTCA surface among CR, C.V, and M.O dyes.


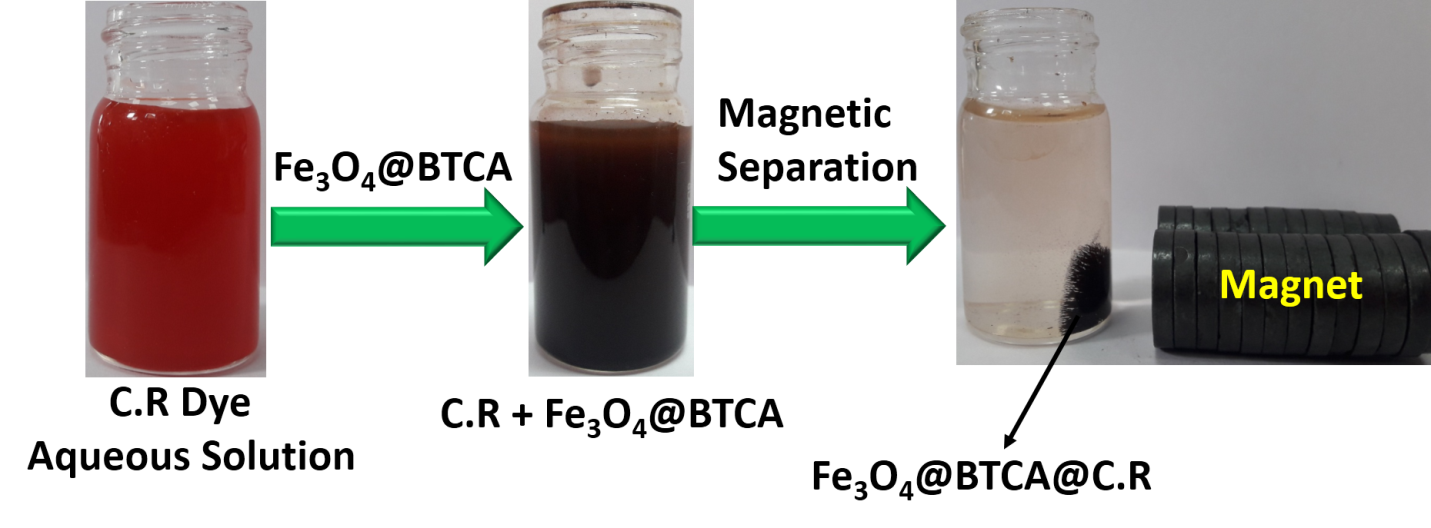


**Figure S10.** Magnetic separation of C.R loaded Fe_3_O_4_@BTCA material from an aqueous solution of C.R dye.


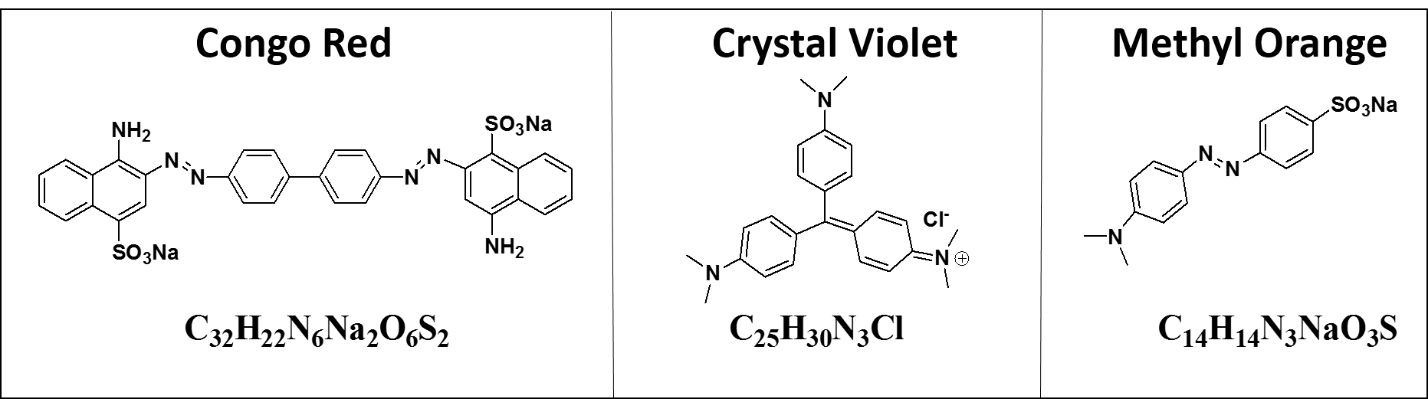


**Figure S11.** Molecular structure of Congo Red, Crystal Violet and Methyl Orange.

**Table S1.** Pore size, pore volume and surface area of Fe_3_O4 and Fe_3_O_4_@BTCA.

| **Sample** | **Pore size (nm)** | **Pore volume (cm^3^/g)** | **Surface area (m^2^/g)** |
| --- | --- | --- | --- |
| Fe_3_O_4_ | 12.408 | 0.171 | 46.446 |
| Fe_3_O_4_@BTCA | 12.313 | 0.280 | 72.347 |

**Table S2.** **A)** Fitting results of adsorption isotherm data with the Langmuir and Freundlich model for C.R dye, **B)** First and second-order Kinetic Parameters for the adsorption of CR on Fe_3_O_4_@BTCA surface

| **A. Adsorption Isotherm** | | | | | | | |
| --- | --- | --- | --- | --- | --- | --- | --- |
| **Langmuir** | | | | | **Freundlich** | | |
| K_L_ (L mg^-1^) | q_max_(mg g^-1^) | | R^2^ | | K_L_(L g^-1^) | n | R^2^ |
| 0.2173 | 729 | | 0.9837 | | 150 | 1.9596 | 0.8722 |
| **B. Kinetic Model** | | | | | | | |
| **Pseudo-1^st^-order** | | | | **Pseudo-2^nd^-order** | | | |
| q_e_ (mg/g) | K_l_ (min^-1^) | R^2^ | | q_e_ (mg/g) | | K_l_(g.mg^-1^min^-1^) | R^2^ |
| 204 | 0.3134 | 0.8781 | | 212 | | 0.0021 | 0.9757 |

**Table S3.** Comparison of adsorption capacities of various Fe_3_O_4_ based NPs (Ref. in the main manuscript) with Fe_3_O_4_@BTCA towards C.R dye.

| **Sample** | **Adsorption capacity (mg/g)** | **Ref** |
| --- | --- | --- |
| Fe_3_O_4_  Fe_3_O_4_/Coke  Fe_3_O_4_@TiO_2_@GO  Fe_3_O_4_/Carbon membrane  GO/Fe_3_O_4_/PEI  Fe_3_O_4_@NiO  Fe_3_O_4_@APTES  Fe_3_O_4_/Bi_2_O_3_  MWCNTs/Fe_3_O_4_/PANI  **Fe_3_O_4_@BTCA** | 28.46  56.19  89.95  48.10  574  128  118  92.24  417  **630** | 62  63  45  64  38  47  39  46  40  **This work** |
